# Monogenic disease analysis establishes that fetal insulin accounts for half of human fetal growth

**DOI:** 10.1172/JCI165402

**Published:** 2023-03-15

**Authors:** Alice E. Hughes, Elisa De Franco, Rachel M. Freathy, Fetal Insulin and Growth Consortium, Sarah E. Flanagan, Andrew T. Hattersley

**Affiliations:** 1 Faculty of Health and Life Sciences, University of Exeter Medical School, Exeter, United Kingdom.; 2 The Fetal Insulin and Growth Consortium is detailed in Supplemental Acknowledgments.

**Keywords:** Development, Endocrinology, Beta cells, Insulin, Obstetrics/gynecology

**To the Editor:** Extremes in birth weight are associated with adverse pregnancy outcomes and long-term risk of cardiometabolic disease. Fetal insulin has long been recognized as an important regulator of fetal growth, but the overall contribution of fetal insulin to birth weight in humans has not been quantified. Single-gene mutations resulting in absent fetal insulin secretion provide a unique opportunity to study the effects of fetal insulin on birth weight in humans. We sought to quantify the role of fetal insulin in fetal growth by studying birth weights in individuals without fetal insulin, either due to recessive loss-of-function mutations in the *INS* gene or pancreatic agenesis (Supplemental Methods and Supplemental Tables 1 and 2 for clinical details and genetics, respectively; supplemental material available online with this article; https://doi.org/10.1172/JCI165402DS1). We also investigated whether reduced insulin-mediated fetal growth affected postnatal growth once insulin was replaced. The study was approved by the Wales Research Ethics Committee (17/WA/0327). *P* values of less than 0.05 were considered statistically significant, and specific statistical tests are detailed in Figure 1.

## In the absence of fetal insulin birth weight is halved in humans.

**Figure 1:**
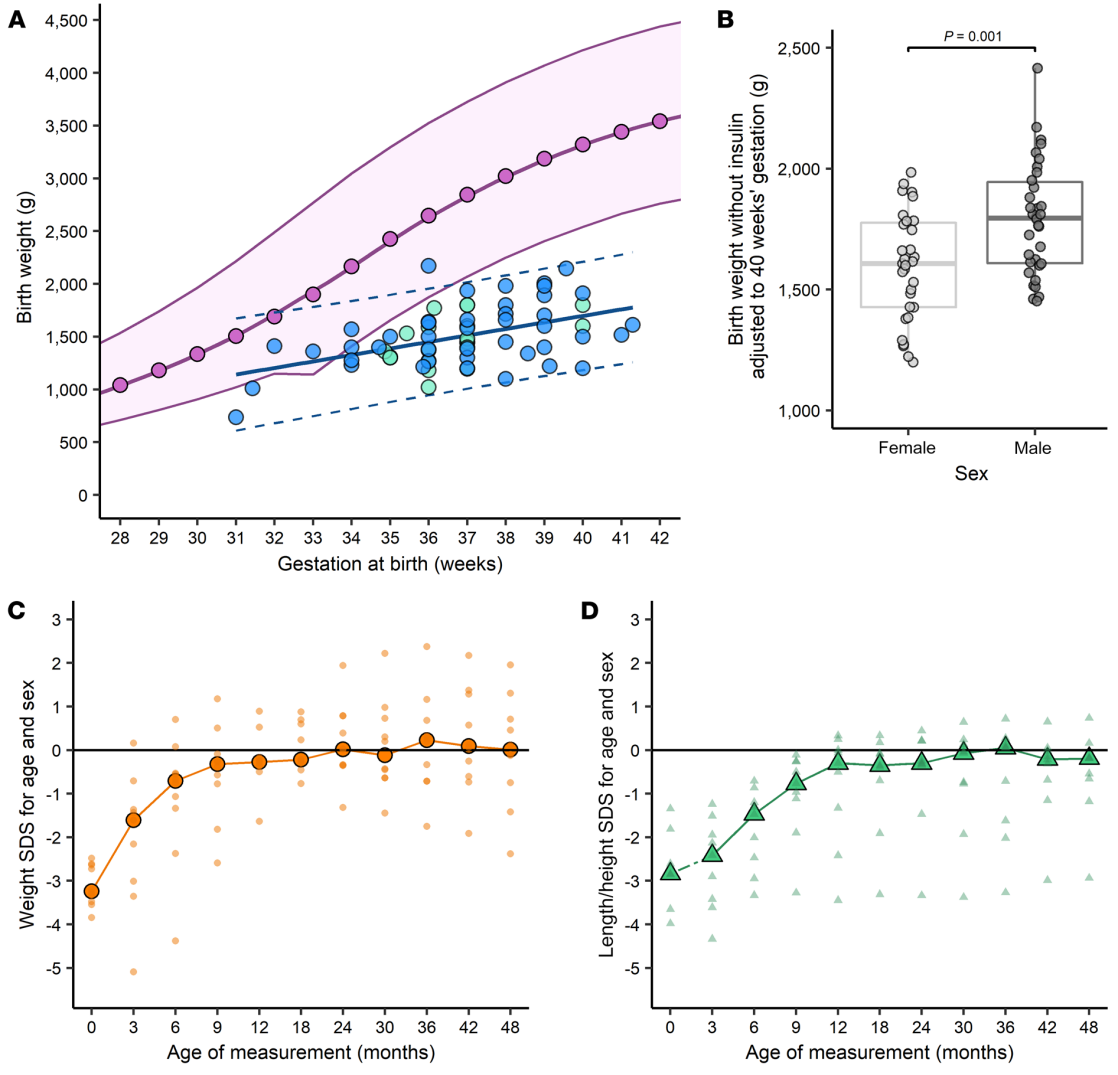
The effect of absent fetal insulin on pre- and postnatal growth in humans. (**A**) Birth weights in individuals without fetal insulin. Light green, *INS* (*n* = 21); blue, pancreatic agenesis (*n* = 43). Normal growth median, dark pink; the shaded pink area indicates ±2 SDs (INTERGROWTH-21^st^, refs. 1, 8). The relationship between birth weight without fetal insulin and gestational age is shown by the line-of-best fit (solid blue) and 95% prediction intervals (dashed blue) from a univariable linear regression model. (**B**) Adjusted birth weight (40 weeks) in the absence of insulin in female (light gray, *n* = 31) and male (dark gray, *n* = 33) individuals. Center line, median; box limits, IQR; whiskers, 1.5 × IQR. Unpaired, 2-tailed Student’s *t* test was used. (**C** and **D**) Growth in first 4 years following insulin treatment in individuals with a recessive *INS* mutation. (**C**) Weight (orange circles, *n* = 10) and (**D**) length/height (green triangles, *n* = 7 birth, *n* = 9 postnatal), with large symbols showing the median. Measurements were standardized (SDS) for sex and gestational age/age (9). Absent data points for individuals were approximated from available data using linear interpolation.

There was a substantial, global reduction in fetal growth in the absence of fetal insulin (Figure 1A and Supplemental Tables 1 and 3). Mean birth weight adjusted to 40 weeks’ gestation was 51% of normal birth weight (1,697 g [95% CI, 1,586–1,808 g] vs. 3,320 g [50th percentile at 40 weeks]) (1). Median birth length was greatly reduced (Supplemental Table 1) and 12% lower than normal (adjusted 43.7 cm [95% CI, 40.9–46.5 cm] vs. 49.6 cm at 40 weeks) (1).

Female individuals without insulin were 196 g (95% CI, 80–312 g) lighter than male individuals, indicating that the sexual dimorphism in birth weight is accounted for by noninsulin-mediated fetal growth (Figure 1B).

## Deficient insulin-mediated growth in utero is accompanied by rapid postnatal catch-up growth once insulin is replaced.

In utero growth restraint due to absent fetal insulin secretion did not persist postnatally. In individuals with loss-of-function *INS* mutations, after birth there was evidence of rapid, early catch-up growth of weight and length (Figure 1, C and D). Compared with birth size, there was a median gain of 2.97 SDS (IQR, 2.29 to 3.07 SDS) in weight and 2.11 SDS (IQR, –0.58 to 3.68 SDS) in length. Most recently available weight (*n* = 16) and height (*n* = 15) after the age of 2 years were within the normal ranges (Supplemental Table 4).

This study utilizing human monogenic disease as a model of absent fetal insulin has provided unique insights into the physiology of early growth.

Insulin-mediated fetal growth in humans contributes approximately 49% to birth weight at term, which is highest out of all species studied (2). Absent fetal insulin also reduced birth length, but its greater effect on weight confirms its main effects relate to fetal fat deposition. The high contribution of fetal insulin-mediated growth to birth weight could explain why, at birth, humans have a higher proportion of body fat compared with other species (3). This high proportion of fat at birth could confer a survival advantage, as lipids provide an efficient and vital fuel for the developing, large human brain (4). The relatively long length of gestation in humans could also result in a longer period of exposure to fetal insulin. We observed birth weight without fetal insulin to deviate further from the normal range as pregnancy progressed (Figure 1A), indicating that insulin-mediated growth becomes more important later in pregnancy.

A key regulator of fetal insulin-mediated growth is maternal glucose. Maternal glycemia has a substantial effect on birth weight (1 mmol/L higher maternal fasting glucose raises birth weight by 301 g; ref. 5). Similar to the situation in which insulin is replaced after birth following in utero deficiency, the effect of maternal glycemia is transient and rapidly lost in the first year of life, with catch-up and catch-down growth (6). In contrast, the lower birth weight in female individuals does not appear to have its origins in fetal insulin-mediated growth, and catch-up is not observed

Rapid catch-up growth once insulin is replaced in individuals with *INS* loss-of-function mutations is in marked contrast to the postnatal growth failure of those with severe insulin resistance secondary to biallelic mutations in the insulin receptor gene *INSR* (Donohue syndrome), despite similarly low birth weight (7). This is likely to reflect that, postnatally, it is not possible to correct the tissue insulin resistance in these individuals.

In conclusion, monogenic diseases resulting in absent fetal insulin have enabled us to answer fundamental questions about early growth in humans. We have used a monogenic human knockout of insulin to show that absence of fetal insulin reduces birth weight by approximately half and postnatally, there is rapid catch-up in weight and length. This establishes that insulin-mediated and noninsulin-mediated growth are equally important in humans. Whether other key modulators of fetal growth apart from maternal glucose act through fetal insulin is uncertain. In the future, all studies looking at fetal growth should determine whether insulin- or noninsulin-mediated growth are impacted, because the short- and long-term outcomes are likely to be different.

## Supplementary Material

Supplemental data

## Version 1 02/21/2023

In-Press Preview

## Version 2 03/15/2023

Electronic publication
