# Supplementary material for: Monogenic disease analysis establishes that fetal insulin accounts for half of human fetal growth
Source: J Clin Invest. 2023 Mar 15;133(6):e165402. doi: 10.1172/JCI165402 (PMC10014100; doi:10.1172/JCI165402)
Supplement: Supplemental data [file jci-133-165402-s155.pdf]

Supplemental material for "Monogenic disease establishes that fetal insulin accounts for half of human fetal growth"

Supplemental note

Members of the Fetal Insulin and Growth Consortium

Name	Affiliation(s)
Adnan Al Shaikh	King Saud bin Abdulaziz University for Health Sciences, King Abdullah International Medical Research Center, King Abdulaziz Medical City, Jeddah, Saudi Arabia
Dorothee Deiss	Medicover Berlin Mitte, Center for Pediatric and Adult Diabetology and Endocrinology, Berlin, Germany
Hüseyin Demirbilek	Department of Paediatric Endocrinology, Hacettepe University Faculty of Medicine, Ankara, Turkey
Sian Ellard	Institute of Biomedical and Clinical Science, University of Exeter Medical School, Exeter, U.K.<br>Exeter Genomics Laboratory, Royal Devon and Exeter Hospitals NHS Foundation Trust, Exeter, U.K.
Koumudi Godbole	Deenanath Mangeshkar Hospital and Research Center, Pune, India
Shaun R Gorman	Bradford Teaching Hospitals NHS Foundation Trust, Bradford, U.K.
Jan-Åke Hammersjö	Department of Pediatrics, Västervik Hospital, Sweden
Jennifer Harrington	Department of Pediatrics, Hospital for Sick Children, University of Toronto, Toronto, Canada
Jayne A L Houghton	Institute of Biomedical and Clinical Science, University of Exeter Medical School, Exeter, U.K.<br>Exeter Genomics Laboratory, Royal Devon and Exeter Hospitals NHS Foundation Trust, Exeter, U.K.
Stanislava Kloušková	Department of Paediatrics, 2nd Faculty of Medicine, Charles University in Prague and University Hospital Motol, Prague, Czech Republic
Selim Kurtoğlu	Erciyes University Faculty of Medicine Department of Pediatric Endocrinology, Kayseri, Turkey
Nuala P Murphy	Children's Health Ireland at Temple St, Dublin and School of Medicine, University College Dublin, Ireland
Michele A O'Connell	Dept of Endocrinology and Diabetes, The Royal Children's Hospital Melbourne, Australia &<br>Centre for Hormone Research, Murdoch Children's Research Institute, Melbourne, Australia
Hasan Önal	Health Sciences University, Başakşehir Çam ve Sakura Şehir Hastanesi, Department of Pediatric Endocrinology and Metabolism, Istanbul, Turkey
Kashyap A Patel	Institute of Biomedical and Clinical Science, University of Exeter of Exeter Medical School, Exeter, U.K.
Klemens Raile	Department of Paediatric Endocrinology and Diabetology, Charité - University Medicine Berlin, Berlin, Germany

Maryam Razzaghy-Azar	Metabolic Disorders Research Center, Endocrinology and Metabolism Molecular-Cellular Sciences Institute, Tehran University of Medical Sciences, Tehran, Iran & Hazrat Aliasghar Children's Hospital, Iran University of Medical Sciences, Tehran, Iran
Zdeněk Šumník	Department of Paediatrics, 2nd Faculty of Medicine, Charles University in Prague and University Hospital Motol, Prague, Czech Republic

Study acknowledgments

The authors are grateful to the participants and their families and acknowledge the genetic testing performed by Exeter Genomics Laboratory. This research was funded in whole, or in part, through grants from the Wellcome Trust to AEH (GW4 Clinical Academic Training PhD Fellowship, WT203918), RMF (Senior Research Fellowship, WT220390), and SEF (Senior Research Fellowship, WT223187), and Diabetes UK to EDF (RD Lawrence Fellowship, 19/005971). ATH is employed as a core member of staff within the National Institute for Health Research (NIHR) Exeter Clinical Research Facility (228). This work was supported by the NIHR Exeter Biomedical Research Centre.

The funders had no role in study design; data collection, analysis or interpretation; or manuscript preparation. The views expressed are the authors’ and not necessarily those of any funder.

Supplemental methods

Identification of individuals with absent fetal insulin secretion

Individuals with recessive mutations in the *INS* gene (detected by targeted Sanger sequencing) or a mutation in a gene known to cause pancreatic agenesis (detected by targeted next generation sequencing as previously described (1)) were identified from an international, multi-ethnic cohort referred to the Exeter Genomics Laboratory for genetic diagnostic testing of neonatal diabetes.

For this study, all individuals had permanent neonatal diabetes requiring insulin treatment that was recognized in the first seven days of life. Individuals with a mutation associated with pancreatic agenesis additionally had evidence of exocrine insufficiency (clinical or biochemical). All individuals needed an available gestational age, sex and weight at birth, leaving 21 individuals with recessive mutations in the insulin gene (*INS*) and 43 individuals with pancreatic agenesis (*CNOT1* [n = 2], *GATA6* [n = 20], *PDX1* [n = 3] or *PTF1A* [n = 19]) included in the final cohort. Follow-up of growth after birth was restricted to those with a recessive *INS* mutation (total n=10), since pancreatic exocrine insufficiency causes malabsorption and may have impacted on growth (2).

Collection of clinical data

Clinical details were provided by referring clinicians. For the postnatal growth follow-up data, we collected serial measures of weight and length/height until at least 4 years of age (where possible). We also requested a most recent weight and height measurement, HbA1c and daily insulin dose. For two individuals, we used measurements at referral (age 11 and 22 years) as the most recently available measurement. All data was routinely collected in a clinical practice setting.

Standardization of anthropometric measurements

Birth weight (n=64) and length (n=17) were standardized for sex and gestational age at birth in weeks and days using the INTERGROWTH-21st standards (3) and studied as standard deviation scores (SDS). Postnatal weight and length/height were standardized for sex and age at measurement (in months and weeks for the first 12 months, then months thereafter) using the WHO Child Growth Standards (4) with correction for gestational age at birth until the age of four years. There were 10 individuals with serial weight measurements and 9 individuals with serial length/height measurements (7 of whom had a corresponding birth length) that could be combined for analysis. We approximated the measurements within each individual to exact three-monthly windows for the first year of life (3, 6, 9 and 12 months) and six-monthly intervals for the next three years of life (18, 24, 30, 36, 42 and 48 months) using a linear interpolation. Most recently available weight (n=16) and height (n=15) were standardized for sex and age of measurement using the UK-WHO/British 1990 Growth Reference (5) since it is integrated with the aforementioned WHO Child Growth Standards and provides standardization for age and sex up until 18 years of age. Most recently available weights and heights in adults were standardized to an age of 18 years.

Statistics

Data were summarized as n (%) for categorical data (sex, ethnic ancestry, parental consanguinity, congenital anomalies), medians and interquartile range (IQR) for non-normally distributed data (time to diagnosis of neonatal diabetes, gestational age at birth, birth length, weight SDS and length/height SDS) and mean and 95% confidence interval (CI) for normally distributed data (birth weight).

The relationship between birth weight and birth length without fetal insulin and gestational age were modelled using a univariable linear regression and prediction intervals and measures adjusted to 40 weeks' gestation were calculated from this model.

P values <0.05 were considered statistically significant. All tests of statistical significance were two-tailed. All analyses were performed in R version 3.6.2 (R Foundation for Statistical

Computing) or Stata version 16.0 (StataCorp, College Station, TX, U.S.A.). Figures were produced in R version 3.6.2 using the ggplot package (6).

Study approval

Written consent from participants (or their responsible guardians, where applicable) for use of their samples and clinical information for research was obtained. Samples and clinical information is stored securely in the Genetic Beta Cell Research Bank (https://www.diabetesgenes.org/current-research/genetic-beta-cell-research-bank/), approved by the Wales Research Ethics Committee (Reference 17/WA/0327).

Supplemental Tables

Supplemental Table 1

Table 1. Clinical characteristics at birth for individuals included in analyses of birth weight without fetal insulin.

Characteristic	Recessive *INS* mutations (n = 21)	Pancreatic agenesis (n = 43)[A]	*P* value for comparison between groups[B]	Whole cohort (n = 64)
**Females (%)**	10 (48)	21 (49)	0.93	31 (48)
**Reported ethnicity (%)**	African 0 (0)<br>Arabic 14 (67)<br>Asian Indian 2 (10)<br>European 3 (14)<br>Mixed 0 (0)<br>Unknown 2 (10)	African 1 (2)<br>Arabic 16 (37)<br>Asian Indian 0 (0)<br>European 17 (40)<br>Mixed 1 (2)<br>Unknown 8 (19)	0.03	African 1 (2)<br>Arabic 30 (47)<br>Asian Indian 2 (3)<br>European 20 (31)<br>Mixed 1 (2)<br>Unknown 10 (16)
**Parental consanguinity (%)**	Yes 17 (81)<br>No 1 (5)<br>Not known 3 (14)	Yes 17 (40)<br>No 23 (53)<br>Not known 3 (7)	2.1×10^{-4}	Yes 24 (38)<br>No 34 (53)<br>Not known 6 (9)
**Presence of congenital anomaly (%)**	Yes 0 (0)<br>No 21 (100)	Yes 24 (56)<br>No 19 (44)	4.0×10^{-6}	Yes 24 (38)<br>No 40 (63)
**Time to diagnosis of PNDM in days (IQR)**	1 (1 to 5)	1 (0 to 2)	0.21	1 (0 to 2)
**Gestational age at birth in weeks (IQR)**	36 (36 to 37)	37 (36 to 39)	0.26	37 (36 to 38)
**Birth weight in grams (95% CI)**	1455 (1354 to 1556)	1523 (1427 to 1618)	0.38	1501 (1430 to 1571)
**Birth weight SDS (IQR)[C]**	-3.17 (-3.48 to -2.64)	-3.11 (-3.56 to -2.49)	0.81	-3.11 (-3.53 to -2.60)
**Birth length in cm (IQR)[C,D]**	42 (39 to 44)	41 (39.5 to 41)	0.41	41 (39.5 to 43.5)
**Birth length SDS (IQR)[C,D]**	-2.84 (-3.65 to -1.55)	-3.29 (-3.62 to -2.45)	0.46	-2.86 (-3.62 to -1.90)

Data are presented as counts with percentages of the group, mean with 95% CI, or medians with IQR as appropriate.

[A]Individuals with pancreatic agenesis had a mutation in *CNOT1* (n = 2), *GATA6* (n = 20), *PDX1* (n = 3) or *PTF1A* (n = 18). [B]Characteristics were compared between the two groups using Pearson's X2 or Fisher's exact test (categorical data), unpaired T-tests (continuous, normally distributed data) or Mann-Whitney U tests (continuous, non-normally

distributed data) where appropriate. [C]Weight and length standard deviation scores (SDS) for sex and gestational age were calculated using the INTERGROWTH-21st standards . [D]Birth length was available for 11 individuals with a homozygous *INS* mutation and 6 individuals with pancreatic agenesis. CI = confidence interval; IQR = interquartile range; PNDM = permanent neonatal diabetes; SDS = standard deviation score.

Supplemental Table 2. Mutation details for individuals with recessive *INS* mutations and pancreatic agenesis included in birth weight cohort.

Gene	Mutation type	Zygosity	Nucleotide change	Mutation name	Number with mutation (N=64)	Reference to papers where participants with mutation have previously been reported
INS	Deletion affecting regulatory region	Homozygous	c.-366_-343del	p.?	1	Garin et al. (7)
INS	Deletion	Homozygous	c.-370-?_186+?del	p.(Met1_Gln62del)	2	Garin et al. (7), Raile et al. (8)
INS	Deletion	Homozygous	c.(?_-1)_(333+1_?)del	p.(Met1_Asn110del)	4	-
INS	Nonsense	Homozygous	c.136C>T	p.(Arg46*)	2	-
INS	Nonsense	Homozygous	c.184C>T	p.(Gln62*)	1	Garin et al. (7)
INS	Promoter	Homozygous	c.-331C>G	p.?	5	Garin et al. (7), Al Shaikh et al. (9), Demiral et al. (10)
INS	Splice site	Homozygous	c.188-15G>A	p.?	4	-
INS	Start-loss	Homozygous	c.3G>A	p.(Met1?)	1	Garin et al. (7)
INS	Start-loss	Homozygous	c.3G>T	p.(Met1?)	1	Garin et al. (7)
CNOT1	Missense	Heterozygous	c.1603G>A	p.(Arg535Cys)	2	De Franco et al. (11),<br><br>Hilbrands et al. (12)
GATA6	Deletion	Heterozygous	c.(?_-1)_(1788+1_?)del	p.?	1	-
GATA6	Deletion	Heterozygous	c.(?-265)_(1135_?)del	p.?	1	-
GATA6	Frameshift	Heterozygous	c.701del	p.(Pro234fs)	1	Lango-Allen et al. (13)
GATA6	Frameshift	Heterozygous	c.744del	p.(Pro249fs)	1	-

GATA6	Frameshift	Heterozygous	c.1036_1042del	p.(Thr346fs)	1	De Franco et al. (14)
GATA6	Frameshift	Heterozygous	c.1108_1121dup	p.(Glu375fs)	1	Lango-Allen et al. (13)
GATA6	Frameshift	Heterozygous	c.1448_1455del	p.(Met483fs)	1	Lango-Allen et al. (13)
GATA6	Missense	Heterozygous	c.1354A>G	p.(Thr452Ala)	1	De Franco et al. (14)
GATA6	Missense	Heterozygous	c.1367G>A	p.(Arg456His)	1	Lango-Allen et al. (13), Balasubramanian et al. (15)
GATA6	Missense	Heterozygous	c.1369A>G	p.(Arg457Gly)	1	-
GATA6	Missense	Heterozygous	c.1396A>G	p.(Asn466Asp)	1	Lango-Allen et al. (13)
GATA6	Missense	Heterozygous	c.1399G>A	p.(Ala467Thr)	1	Lango-Allen et al. (13)
GATA6	Nonsense	Heterozygous	c.969C>A	p.(Tyr323*)	2	De Franco et al. (14)
GATA6	Nonsense	Heterozygous	c.1242C>A	p.(Cys414*)	1	Tuhan et al. (16)
GATA6	Splice site	Heterozygous	c.1303-2A>G	p.?	1	-
GATA6	Splice site	Heterozygous	c.1303-10C>G	p.?	1	Lango-Allen et al. (13)
GATA6	Splice site	Heterozygous	c.1429-41_1441del	p.?	1	De Franco et al. (14)
GATA6	Splice site	Heterozygous	c.1516+1G>C	p.?	1	Lango-Allen et al. (13), Wintergerst et al. (17)

GATA6	Splice site	Heterozygous	c.1516+4A>G	p.?	1	Lango-Allen et al. (13),<br><br>Barbarini et al. (18)
PDX1	Missense	Homozygous	c.478C>A	p.(Glu160Lys)	1	-
PDX1	Missense	Homozygous	c.524G>A	p.(Arg175His)	1	-
PDX1	Missense	Homozygous	c.524G>T	p.(Arg175Leu)	1	-
PTF1A	Enhancer	Homozygous	g.23508305A>G	p.?	1	Weedon et al. (19)
PTF1A	Enhancer	Homozygous	g.23508363A>G	p.?	5	Evliyaoğlu et al. (20)
PTF1A	Enhancer	Homozygous	g.23508365A>G	p.?	1	Weedon et al. (19)
PTF1A	Enhancer	Homozygous	g.23508437A>G	p.?	6	Weedon et al. (19)
PTF1A	Frameshift/enhancer	Compound heterozygous	c.437_462del/g.23508442A>G	p.(Ala146fs)/p.?	1	Gabbay et al. (21)
PTF1A	Missense	Homozygous	c.571C>A	p.(Pro191Thr)	3	Houghton et al. (22)
PTF1A	Start-loss	Homozygous	c.1A>G	p.(Met1?)	1	-

Supplemental Table 3. Birth weights in cohort by different phenotypic/genotypic groups.

Group	Mean birth weight, g (95% CI)	Mean birth weight adjusted to 40 weeks' gestation, g (95% CI)[A]	Median birth weight SDS (IQR)[B]
Whole cohort (N=64)	1501 (1430 to 1571)	1696 (1585 to 1806)	-3.11 (-3.53 to -2.59)
INS mutation (N=21)	1455 (1354 to 1556)	1730 (1483 to 1976)	-3.17 (-3.48 to -2.64)
Pancreatic agenesis (N=43)	1523 (1427 to 1618)	1698 (1564 to 1833)	-3.11 (-3.56 to -2.49)
Isolated *INS* mutation or pancreatic agenesis (N=40)	1505 (1427 to 1583)	1693 (1542 to 1844)	-3.13 (-3.43 to -2.63)
Isolated pancreatic agenesis (N=19)	1561 (1200 to 1980)	1671 (1454 to 1887)	-3.12 (-3.29 to -2.58)
Additional structural anomaly present[C] (N=24)	1492 (1346 to 1639)	1704 (1504 to 1904)	-2.96 (-3.69 to -2.44)
Coding *INS* mutation[D] (N= 9)	1456 (1359 to 1554)	1650 (1438 to 1862)	-2.74 (-3.17 to -2.61)
Non-coding *INS* mutation[E] (N=12)	1455 (1278 to 1631)	1851 (1379 to 2323)	-3.30 (-3.53 to -2.76)

[A]Calculated from a univariable linear regression model including birth weight as the dependent variable and gestational age at birth in weeks as the independent variable. [B]Weight standard deviation scores (SDS) for sex and gestational age were calculated using the INTERGROWTH-21st standards (3). [C]Cardiac, brain, gastrointestinal. [D]Nonsense or deletion affecting coding region of gene. [E]Splice site or promoter region. CI = confidence interval; IQR = interquartile range; SDS = standard deviation score

Supplemental Table 4. Most recently available clinical measurements for 16 individuals of original birth weight cohort with recessive *INS* mutations. Data are presented as medians with IQR.

Age of measurement (years)	Weight SDS for age and sex[A]	Height SDS for age and sex[A,B]	HbA1c (mmol/mol)	Insulin dose (units/kg)
14 (7 to 18)	0.19 (-0.67 to 1.33)	-0.78 (-1.78 to -0.19)	58.5 (55.1 to 78.1)	0.97 (0.78 to 1.07)

[A]Calculated using the WHO Child Growth Standards (4) up until the age of four years, then the British 1990 Reference (UK-WHO) (5) up until the age of 18 years. Where the most recent measurement was after the age of 18 years, we standardized to 18 years. [B]Available for 15 out of 16 individuals. IQR = interquartile range

Supplemental material References

1. Ellard S et al. Improved genetic testing for monogenic diabetes using targeted next-generation sequencing. *Diabetologia* 2013;56(9):1958–1963.

2. Bronstein MN et al. Pancreatic insufficiency, growth, and nutrition in infants identified by newborn screening as having cystic fibrosis. *JPediatr* 1992;120(4, Part 1):533–540.

3. Villar J et al. International standards for newborn weight, length, and head circumference by gestational age and sex: the Newborn Cross-Sectional Study of the INTERGROWTH-21st Project. *Lancet* 2014;384(9946):857–868.

4. de Onis M. WHO Child Growth Standards based on length/height, weight and age. *Acta Paediatrica* 2006;95(S450):76–85.

5. Cole TJ, Freeman JV, Preece MA. British 1990 growth reference centiles for weight, height, body mass index and head circumference fitted by maximum penalized likelihood. *Stat Med* 1998;17(4):407–429.

6. Wickham H. *ggplot2: Elegant Graphics for Data Analysis*. Springer-Verlag; 2009.

7. Garin I et al. Recessive mutations in the INS gene result in neonatal diabetes through reduced insulin biosynthesis. *Proc Natl Acad Sci U.S.A.* 2010;107(7):3105–3110.

8. Raile K et al. Diabetes caused by insulin gene (INS) deletion: clinical characteristics of homozygous and heterozygous individuals. *Eur J Endocrinol* 2011;165(2):255–260.

9. Shaikh AA, Shirah B, Alzelaye S. A homozygous mutation in the insulin gene (INS) causing autosomal recessive neonatal diabetes in Saudi families. *Ann Pediatr Endocrinol Metab* 2020;25(1):42–45.

10. Demiral M et al. Neonatal diabetes due to homozygous INS gene promoter mutations: Highly variable phenotype, remission and early relapse during the first 3 years of life. *Pediatr Diabetes* 2020;21(7):1169–1175.

11. De Franco E et al. A Specific CNOT1 Mutation Results in a Novel Syndrome of Pancreatic Agenesis and Holoprosencephaly through Impaired Pancreatic and Neurological Development. *Am J Hum Genet* 2019;104(5):985–989.

12. Hilbrands R et al. Pancreas and gallbladder agenesis in a newborn with semilobar holoprosencephaly, a case report. *BMC Med Genet* 2017;18(1):57.

13. Allen HL et al. GATA6 haploinsufficiency causes pancreatic agenesis in humans. *Nat Gen* 2012;44(1):20–22.

14. De Franco E et al. GATA6 Mutations Cause a Broad Phenotypic Spectrum of Diabetes From Pancreatic Agenesis to Adult-Onset Diabetes Without Exocrine Insufficiency. *Diabetes* 2013;62(3):993–997.

15. Balasubramanian M et al. Pancreatic hypoplasia presenting with neonatal diabetes mellitus in association with congenital heart defect and developmental delay. *Am J Med Genet A* 2010;152A(2):340–346.

16. Tuhan H et al. Neonatal diabetes mellitus due to a novel mutation in the GATA6 gene accompanying renal dysfunction: a case report. *Am J Med Genet A* 2015;167A(4):925–927.

17. Wintergerst KA, Hargadon S, Hsiang HY. Continuous subcutaneous insulin infusion in neonatal diabetes mellitus. *Pediatr Diabetes* 2004;5(4):202–206.

18. Barbarini DS et al. Neonatal diabetes mellitus due to pancreas agenesis: a new case report and review of the literature. *Pediatr Diabetes* 2009;10(7):487–491.

19. Weedon MN et al. Recessive mutations in a distal PTF1A enhancer cause isolated pancreatic agenesis. *Nat Genet* 2014;46(1):61–64.

20. Evliyaoğlu O et al. Neonatal Diabetes: Two Cases with Isolated Pancreas Agenesis due to Homozygous PTF1A Enhancer Mutations and One with Developmental Delay, Epilepsy, and Neonatal Diabetes Syndrome due to KCNJ11 Mutation. *J Clin Res Pediatr Endocrinol* 2018;10(2):168–174.

21. Gabbay M, Ellard S, De Franco E, Moisés RS. Pancreatic Agenesis due to Compound Heterozygosity for a Novel Enhancer and Truncating Mutation in the PTF1A Gene. *J Clin Res Pediatr Endocrinol* 2017;9(3):274–277.

22. Houghton JAL et al. Isolated Pancreatic Aplasia Due to a Hypomorphic PTF1A Mutation. *Diabetes* 2016;65(9):2810–2815.
